# Impact of laparoscopic vertical sleeve gastrectomy (LVSG) on lower esophageal sphincter pressure (LESP), lower esophageal sphincter length (LESL) and gastroesophageal reflux disease (GERD) using esophageal function tests (EFTs): a systematic review and meta-analysis

**DOI:** 10.1038/s41366-025-01926-y

**Published:** 2025-10-06

**Authors:** Muhammed Ashraf Memon, Rossita Mohamad Yunus, Khorshed Alam, Zahirul Hoque, Shahjahan Khan

**Affiliations:** 1 School of Mathematics, Physics and Computing and Centre for Health Research, University of Southern Queensland, Toowoomba, QLD Australia; 2 Sunnybank Obesity Center and South East Queensland Surgery (SEQS), Sunnybank, QLD Australia; 3 Mayne Medical School, School of Medicine, University of Queensland, Brisbane, QLD Australia; 4 Faculty of Health Sciences and Medicine, Bond University, Gold Coast, QLD Australia; 5 Faculty of Health and Social Science, Bolton University, Bolton, Lancashire UK; 6 Institute of Mathematical Sciences, Universiti Malaya, Kuala Lumpur, Malaysia; 7 School of Business and Centre for Health Research, University of Southern Queensland, Toowoomba, QLD Australia; 8 School of Mathematics, Physics and Computing, University of Southern Queensland, Toowoomba, QLD Australia; 9 School of Science and Engineering, Asian University of Bangladesh, Dhaka, Bangladesh

**Keywords:** Health sciences, Signs and symptoms

## Abstract

**Background:**

LVSG seems to increase the risk of GERD despite significant weight loss. We compared pre- and postoperative esophageal function test data (in conjunction with the BMI loss) to evaluate the impact of post-LVSG on lower esophageal sphincter pressure (LESP), lower esophageal sphincter length (LESL), and DeMeester Score (DMS).

**Methods:**

Articles analyzing esophageal manometry ±24 h pH-study pre- and post-LVSG were identified using electronic databases from 1999 to 2023. The Critical Appraisal Skills Programme Checklist for Cohort Studies was used for quality assessment. The DerSimonian and Laird random effects model was used for continuous data analysis. Heterogeneity was assessed using the Cochrane Q statistic and *I*^*2*^ index. Leave one out sensitivity analysis was undertaken to assess the robustness and validity of our analysis. Egger’s test was used to evaluate potential publication bias in our meta-analysis.

**Results:**

Nineteen studies totaling 668 patients were evaluated (*F* = 445, *M* = 131). A significant reduction of 3.82 mm Hg in LESP was observed after LVSG based on 16 studies (WMD 3.82, 95% CI 1.74, 5.90; *p* < 0.001, *I*^*2*^ = 88.6%). LESL did not reveal any significant difference between pre- and post-LVSG based on nine studies (WMD 0.05, 95% CI –0.15, 0.26; *p* = 0.625, *I*^*2*^ = 83.1%). DMS showed a significant increase of 11.72 post LVSG based on 12 studies (WMD –11.72, 95% CI –17.15 to –6.30; *p* < 0.001, *I*^*2*^ = 91.5%). Significant BMI loss of 13.26 kg/m^2^ was observed post LVSG based on 12 studies (WMD 13.26, 95% CI 11.65 to 14.88, *Z* = 16.07, *p* < 0.001).

**Conclusions:**

LVSG is associated with a significant decrease in LESP and a significant increase in the DMS post-LVSG, leading to the worsening or development of new GERD symptoms despite significant BMI reduction. The limitations of our meta-analysis include small sample sizes, short follow-up, heterogeneity, lack of data on some confounders and inadequate quality of some studies.

## Introduction

The lower esophageal sphincter (LES), a high-pressure zone (HPZ) at the junction of the esophagus and stomach, is the most effective barrier to prevent reflux of gastric contents into the esophagus at rest [1–3]. It comprises both intrinsic and extrinsic components. The intrinsic component consists of esophageal muscle fibers that are under neurohormonal control. The extrinsic component consists of the crural diaphragm and phrenoesophageal ligament, also known as the Laimer membrane, which arises from the subdiaphragmatic and endothoracic fascia and attaches the esophagus to the diaphragm, providing anatomical support to the LES and additional protection against gastric reflux [1]. The three most important aspects of LES that provide resistance to gastroesophageal reflux include the LES pressure (LESP), total length of LES (LESL), and abdominal length of LES (LESAL) exposed to the positive pressure environment of the abdomen [2]. Failure of any of these three LES components may lead to gastroesophageal reflux disease (GERD) [2, 4]. Research has shown that laparoscopic vertical sleeve gastrectomy (LVSG) leads to anatomical changes at the esophagogastric junction (EGJ), which in turn leads to physiological consequences such as an increased risk of GERD or even the appearance of de novo GERD due to LES dysfunction [5] despite significant weight loss. However, assessing the effect of GERD after LVSG is challenging due to the lack of standardized reporting, varying definitions, and often lack of objective assessment with esophageal function tests (EFTs) such as manometry and 24 h pH study [6]. Therefore, we conducted a systematic literature search and an in-depth review of the effect of LVSG and its impact on LES and subsequent consequences based on the analysis of preoperative and postoperative data on esophageal manometry and a 24 h pH study in patients with morbid obesity.

## Material and methods

### Search strategies and data collection

Electronic databases (Medline, PubMed, EMBASE, Cochrane Register of Systematic Reviews, Science Citation Index) were searched extensively to identify published studies using either conventional manometry (CM) or high-resolution manometry (HRM) and/or 24 h ambulatory pH study pre- and post-LVSG. Identical search terms for each search engine were chosen to optimize and identify all published papers that met the inclusion criteria and to exclude duplicate papers. Search strategies utilized included combinations of “laparoscopy”[MeSH Terms] OR “laparoscopy”[All Fields] OR “laparoscopic”[All Fields]), “gastric sleeve”[All Fields] OR “sleeve gastrectomy” OR “vertical sleeve gastrectomy” [All Fields] “gastroesophageal reflux disease”[All Fields] OR “gastro-oesophageal reflux disease”[All Fields] “weight loss surgery”[All Fields] “bariatric surgery”[All Fields] “manometry”[All Fields] “lower esophageal OR oesophageal sphincter”[All Fields] “esophageal OR oesophageal function”[All Fields] “esophageal OR oesophageal motility disorder”[All Fields] “esophageal OR oesophageal motor disorder”[All Fields] “esophageal OR oesophageal dysmotility”[All Fields] AND “outcomes”[All Fields]. The reference lists of all retrieved articles were examined for additional citations. Two authors (MAM and RMY) conducted a literature search and selected records that met the inclusion criteria. The same two authors extracted data from the selected studies, compiled data for meta-analysis, and prepared the draft article.

### Inclusion criteria

Type of Studies: Cohort studies in full peer-reviewed journals

Publication dates: January 1999 to November 2023

Type of Intervention: LVSG

Type of participants: Adults with morbid obesity (>18 years)

Esophageal physiological test: Conventional or high-resolution manometry ±24 h ambulatory pH study

Size of study: No restrictions

Sample Language: No language restrictions

### Exclusion criteria

Non-human studies, duplicate studies, abstracts, conference articles, opinion pieces, editorial letters, case studies, reviews, and meta-analyses were excluded from the final review.

### Quality assessment

The Critical Appraisal Skill Programme (CASP) for Cohort Study Checklist [7] was used to assess the quality of the included studies (Table 1). The CASP appraisal skills enable researchers to evaluate (a) the study design and understand its suitability for addressing the research question, (b) the measurement and classification of exposure (i.e., LVSG) and outcomes (on LES), considering the reliability, validity, and potential bias in their assessment, and (c) critically analyze the results for both statistical significance and clinical significance to determine the relevance and implications of the findings.

**Table 1:** The Critical Appraisal Skills Programme (CASP) checklist for cohort study.

	Braghetto et al. [16]	Kledi et al. [17]	Burgerhart et al. [18]	Del Genio et al. [19]	Rebecchi et al. [20]	Gorodner et al. [21]	Sioka et al. [22]	Valezi et al. [23]	Coupaye et al. [24]	Ruiz de Angulo et al. [25]	Raj et al. [26]	Castagneto-Gissey et al. [27]	Gemici et al. [28]	Greilsamer et al. [29]	Navarini et al. [30]	Quero et al. [31]	Tolone et al. [32]	Chern et al. [33]	Poggi et al. [34]
**Section A: Are the results of the study valid**																			
1. Did the study address a clearly focused issue?	Y	Y	Y	Y	Y	Y	Y	Y	Y	Y	Y	Y	Y	Y	Y	Y	Y	Y	Y
2. Was the cohort recruited in an acceptable way?	Y	Y	Y	Y	Y	Y	Y	Y	Y	Y	Y	Y	Can’t tell	Y	Y	Y	Y	Y	Can’t tell
3. Was the exposure accurately measured to minimize bias?	Y	Y	Y	Y	Y	Y	Y	Y	Y	Y	Y	Y	Y	Y	Y	Y	Y	Y	Y
4. Was the outcome accurately measured to minimize bias?	Y	Y	Y	Y	Y	Y	Y	Y	Y	Y	Y	Y	Y	Y	Y	Y	N	Y	Y
5. (a) Have the authors identified all important confounding factors?	Can’t tell	Y	Can’t tell	Y	Y	Y	Y	Y	Y	Can’t tell	Y	Y	Can’t tell	Y	Y	Y	Y	Can’t tell	Can’t tell
5. (b) Have they taken account of the confounding factors in the design and/or analysis?	Can’t tell	Y	Can’t tell	Y	Y	Y	Y	Y	Y	Can’t tell	Y	Y	Can’t tell	Y	Y	Y	Y	Can’t tell	Can’t tell
6. (a) Was the follow up of subjects complete enough?	Y	Y	Y	Y	Y	N	Y	Y	Y	Y	Y	Y	Y	Y	Y	Y	Y	Y	Y
6. (b) Was the follow up of subjects long enough?	N	N	N	Y	Y	Y	N	Y	Y	Y	N	Y	N	Y	Y	N	Y	N	Y
**Section B: What are the results?**																			
7. What are the results of this study?	↓ LESP	↓LESP	↓LESP ↑DMS	≡LESP ↓LESL ↑DMS	≡LESP ≡LESL ≡DMS	↓LESP ≡LESL ↑DMS	↓LESP ↓LESL	↓ LESP	↓LESP ↑DMS	↓LESP ↑LESL ↑DMS	≡LESP ≡LESL ↑DMS	≡LESP ≡DMS	↓LESP ≡LESL ↑DMS	↓LESP ↓LESL ↑DMS	↓LESP ↑DMS	↓LESP ↓LESL	≡LESP	≡LESP ≡LESL ≡DMS	↓LESP ↓DMS
8. How precise are the results?	Precise	Precision lacking	Precise	Very precise	Very precise	Very precise	Precise	Precise	Precise	Precise	Precise	Precise	Precise	Precision lacking	Precise	Precise	Precision lacking	Very precise	Very precise
9. Do you believe the results?	Y	Y	Y	Y	N	Y	Y	Y	Y	Y	Y	N	Y	N	Y	Y	Y	Y	Y
**Section C: Will the results help locally?**																			
10. Can the results be applied to the local population?	Y	Y	Y	Y	N	Y	Y	Y	Y	Y	Y	N	Y	Y	Y	Y	Y	Y	Y
11. Do the results of this study fit with other available evidence?	Y	Y	Y	Y	N	Y	Y	Y	Y	Y	Y	N	Y	N	Y	Y	Y	Y	Y
12. What are the implications of this study for practice?	LVSG ↑ GERD risk	LVSG ↑ GERD risk	LVSG ↑ GERD risk	LVSG ↑ GERD risk	LVSG improves GERD	LVSG ↑ GERD risk	LVSG ↑ GERD risk	LVSG ↑ GERD risk	LVSG ↑ GERD risk	LVSG ↑ GERD risk	LVSG ↑ GERD risk	LVSG doesn’t ↑GERD risk	LVSG ↑ GERD risk	LVSG ↑ GERD risk	LVSG ↑ GERD risk	LVSG ↑ GERD risk	LVSG ↑ GERD risk	LVSG ↑ GERD risk	LVSG ↑ GERD risk

*GERD* gastroesophageal reflux disease, *LVSG* laparoscopic vertical sleeve gastrectomy, *N* No, *Y* Yes, ↓ Decrease, ↑ Increase, ≡ Identical to or equivalent.

### Study design

**Fig. 1:**
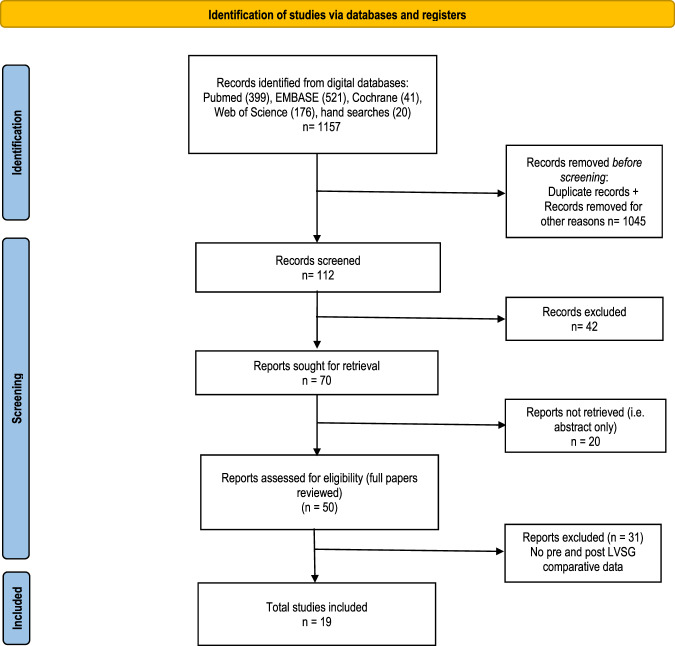
PRISMA flow chart.

After screening the titles and abstracts, articles that fulfilled the inclusion criteria were identified, and their full texts were reviewed (Fig. 1). The data were extracted and stored in Microsoft Excel data Sheets (Microsoft Corporation, Redmond, Washington, USA). The following data were extracted from each article: authors, country of publication, year of publication, number of patients, sex, age, pre-and post LVSG body mass index (BMI), pre- and post-LVSG LESP, pre- and post-LVSG LESL, pre and post-LVSG LESAL and pre- and post-LVSG DeMeester Score (DMS).

### Data extraction (outcome variables)

The primary outcome variables analyzed included pre- and post-LVSG LESP, LESL, and DMS, based on esophageal manometry and 24 h pH study data. We further analyzed pre-and post-LVSG BMI loss. Owing to the lack of availability of data on LESAL, it was excluded from the final analysis.

### Statistical analysis

All included studies underwent qualitative analysis, and a meta-analysis was undertaken for variables for which sufficient data were available. For continuous variables, weighted mean differences (WMD) were used to calculate the effect size using DerSimonian and Laird’s method for the random effects model (REM) [8]. Heterogeneity was assessed using the Cochrane Q statistic and *I*^*2*^ index [9]. To pool continuous data, the mean and standard deviation of each study were required, which were missing in several studies. However, these studies reported the effect size of the trials in terms of median and interquartile range. Using these summary statistics, mean and standard deviation estimates were obtained using the formulas proposed by Hozo et al. [10]^.^ Point estimates of the population effect sizes and forest plots with 95% confidence intervals were produced using the metafor package in R [11, 12]. Similarly, funnel plots were generated using the R package to assess the presence of publication bias [13, 14]. Egger’s test [13] was used to assess potential publication bias. We also undertook “leave-one-out sensitivity analysis” which involved systematically removing one study at a time and re-analyzing our meta-analysis, to assess how much each individual study influences the overall effect estimate and identify potential outliers or influential studies [11]. By comparing the results of the meta-analyses with and without a specific study, we recalculated the pooled results (pooled WMD) and the *I*^2^ statistic to gauge the robustness and stability of our findings. The Pearson correlation coefficient was used to measure the linear correlation between two variables, LVSG and LES [15]. The test of the significance of the population effect size was conducted using the z-statistic. Statistical significance was set at *P* < 0.05.

## Results

**Fig. 2:**
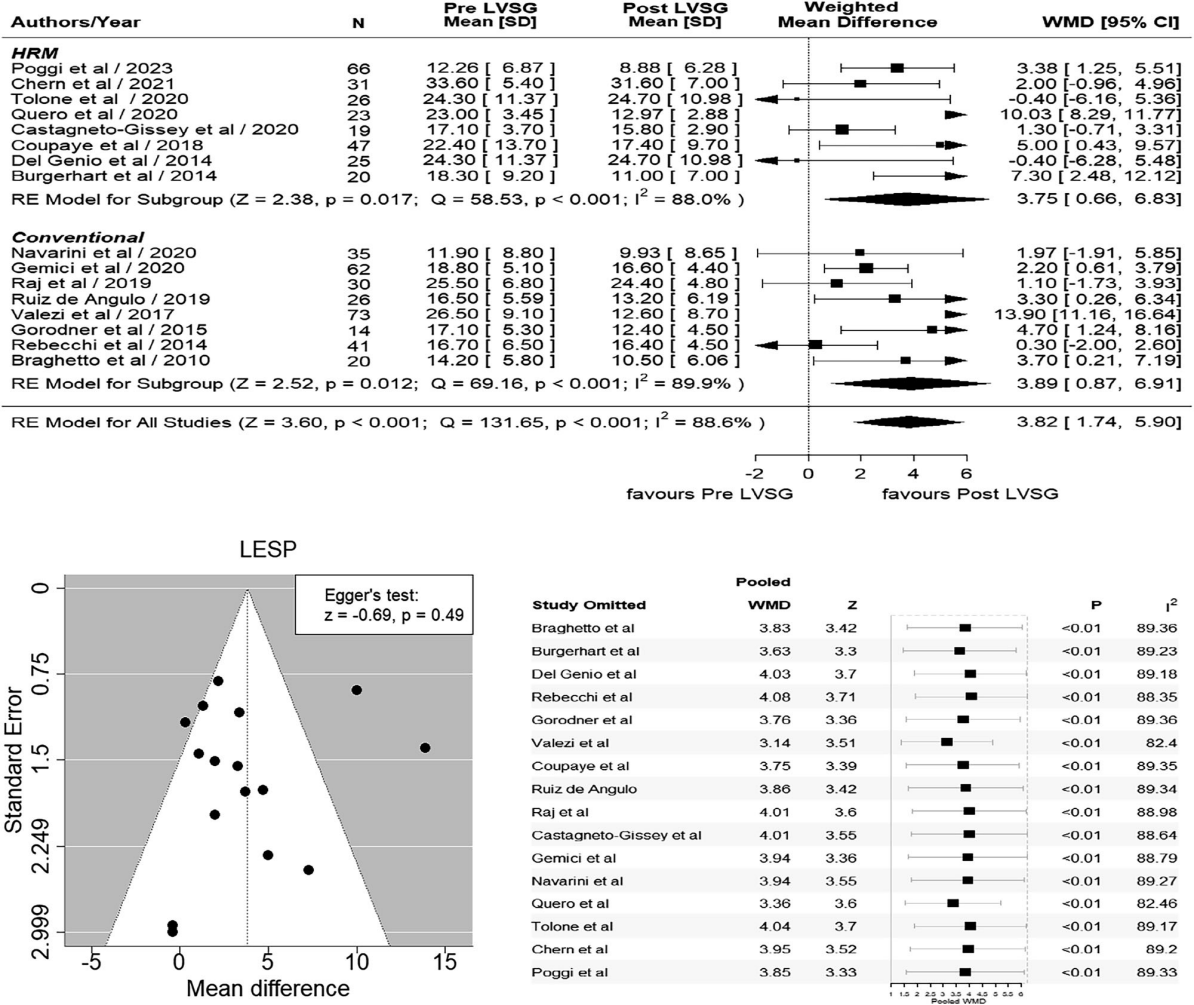
Forrest and funnel plots and sensitivity analysis of LESP (*r* = 0.1). **Forest plot (Top):** Horizontal lines emerging from the square represent 95% confidence intervals (CI) for the study’s result with arrows indicating that a CI extending beyond the boundaries of the graph’s *x* axis and would go beyond the displayed range preventing it from being completely visualized on the plot. A shorter horizontal line means the study’s estimate is more precise. The size of the square is proportional to the weight of that study, which often reflects its sample size or precision. The pooled weighted mean difference (WMD) combines all the MDs of each study using the random effects model with the Der Simonian and Laird method, depicted by the diamond at the bottom represents the overall pooled estimate of the effect from all included studies. The position of the diamond and its width (representing the 95% CI) indicate the overall effect and its precision. Significant reduction was observed in LESP of 3.82 mmHg post LVSG compared to pre LVSG (WMD 3.82, 95% CI 1.74 to 5.90, *Z* = 3.60, *p* < 0.001). **Funnel Plot (bottom left):** Each dot in the plot represents an individual study. The *x*-axis shows the effect size of each study expressed as WMD. The *y*-axis is the standard error and represents the study precision. Larger studies typically have greater precision, whereas smaller studies are less powerful and have lower precision. The overall effect and 95% CIs are denoted by dotted black lines (central and funnel lines, respectively). Significant difference between all studies was noted (*Q* = 131.65, *p* < 0.001, ${I}^{2}$ = 88.6%). The asymmetric distribution of studies around the overall effect indicates the presence of publication bias (study heterogeneity), however, there was no publication bias demonstrated by Egger’s test (*p* = 0.49). **Sensitivity Analysis (bottom right):** In this sensitivity analysis, “a leave one out method” is used, where an individual study is omitted at a time from all studies in this analysis and the weighted effect size and heterogeneity are recalculated to assess the robustness of our analysis. Horizontal lines in the plot represent the 95% CIs for each study, with squares representing the weight of individual studies. The largest decrease in the ${I}^{2}$ value was observed when Valezi et al. was omitted from our analysis. However, our results across multiple iterations i.e., when we removed one data point/individual study did not significantly change the outcome, and the pooled WMD and heterogeneity (>80%) remained relatively unchanged, indicating that the overall conclusions are robust.

**Fig. 3:**
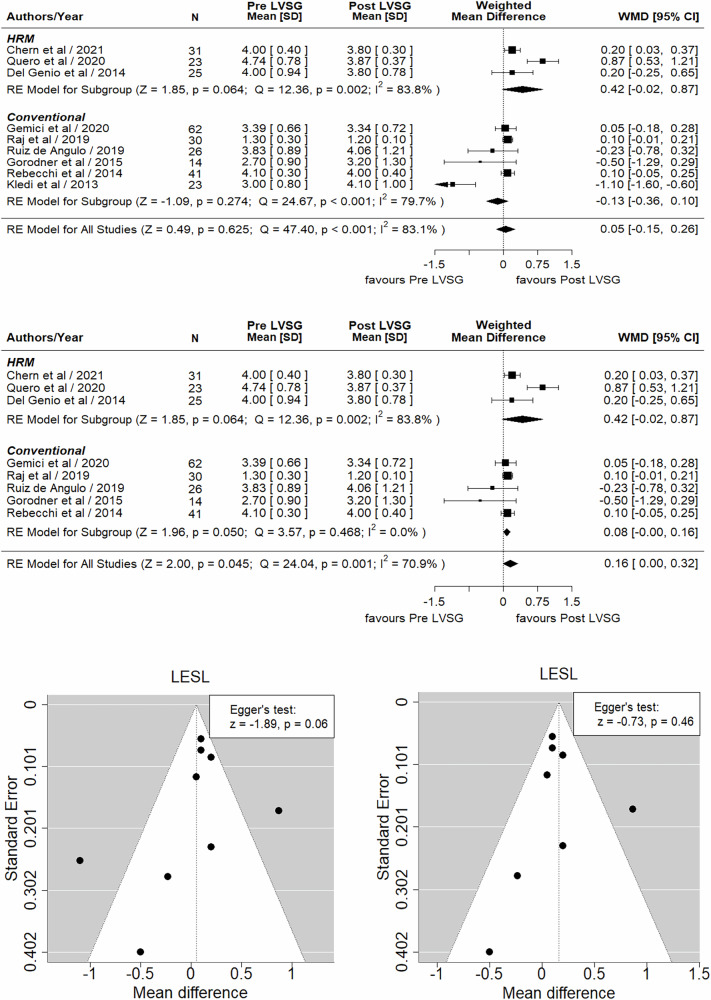
Forrest and funnel plots and sensitivity analysis of LESL (*r* = 0.1). **Forrest Plots with and without Keldi’s study (Top):** There was no significant difference observed in the LESL between post-LVSG and pre-LVSG (WMD 0.05, 95% CI –0.15 to 0.26, *Z* = 0.49, *p* = 0.625). However, with the removal of Keldi’s et al. study, LESL showed significant decrease of 0.16 cm (WMD 0.16, 95% CI 0.00 to 0.32, *Z* = 2.00, *p* = 0.045). Significant difference between all studies was noted (*Q* = 47.40, *p* < 0.001, ${I}^{2}$ = 83.1% with Kledi et al. study and *Q* = 24.04, *p* < 0.001, ${I}^{2}$ = 70.9% without Kledi et al. study). **Funnel Plot with (middle right) and without Keldi’s study (middle left):** The asymmetric distribution of studies around the overall effect indicates the presence of publication bias (study heterogeneity). Egger’s test did confirm publication bias (*p* = 0.06). However, after removing Keldi’s study, although the funnel plot was asymmetrical, there was no publication bias as per Egger’s test (*p* = 0.46). **Sensitivity Analysis (bottom right):** The largest decrease in the ${I}^{2}$ value was observed when Kledi et al. was omitted from our meta-analysis. However, our results across multiple iterations i.e., when we removed one data point/individual study, did not significantly change the outcome, and the pooled WMD and heterogeneity (>70%) remained relatively unchanged, indicating that the overall conclusions are robust.

**Fig. 4:**
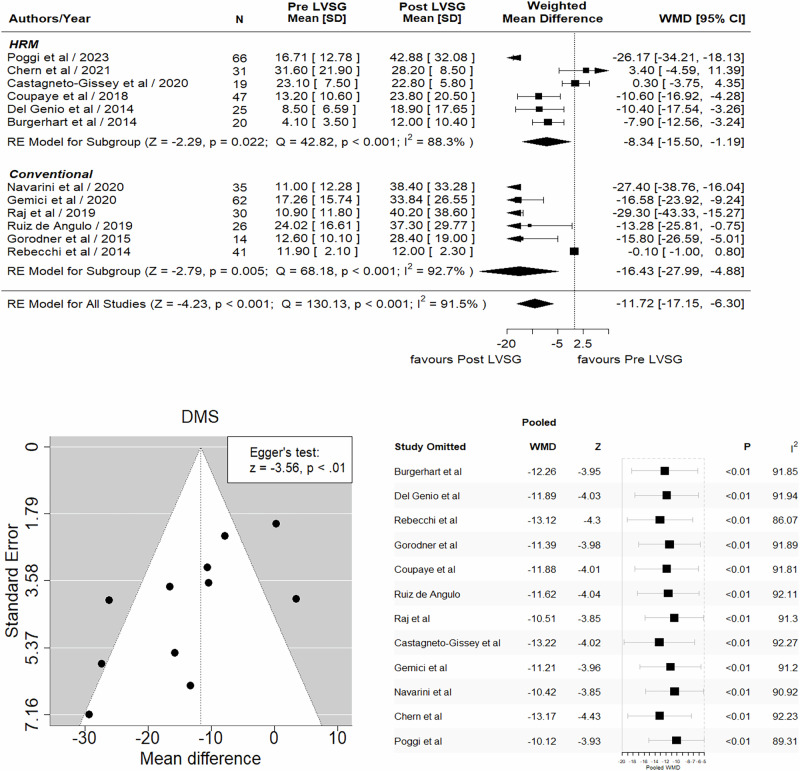
Forrest and funnel plots and sensitivity analysis of DMS (*r* = 0.1). **Forest plot (Top):** Significant increment was observed in DMS of 11.72% post LVSG compared to pre LVSG (WMD –11.72, 95% CI –17.15 to –6.30, *Z* = –4.23, *p* < 0.001). Significant difference between all studies was noted (*Q* = 130.13, *p* < 0.001, ${I}^{2}$ = 91.5%). **Funnel Plot (bottom left):** The asymmetric distribution of studies around the overall effect indicates the presence of publication bias (study heterogeneity). Egger’s test confirmed the publication bias (*p* = <0.01). **Sensitivity Analysis (bottom right):** The value of ${I}^{2}$ remained large when one data point/individual study was removed and did not significantly change the outcome. Furthermore, the pooled WMD and heterogeneity (>80%) remained relatively unchanged, indicating that the overall conclusions are robust.

**Fig. 5:**
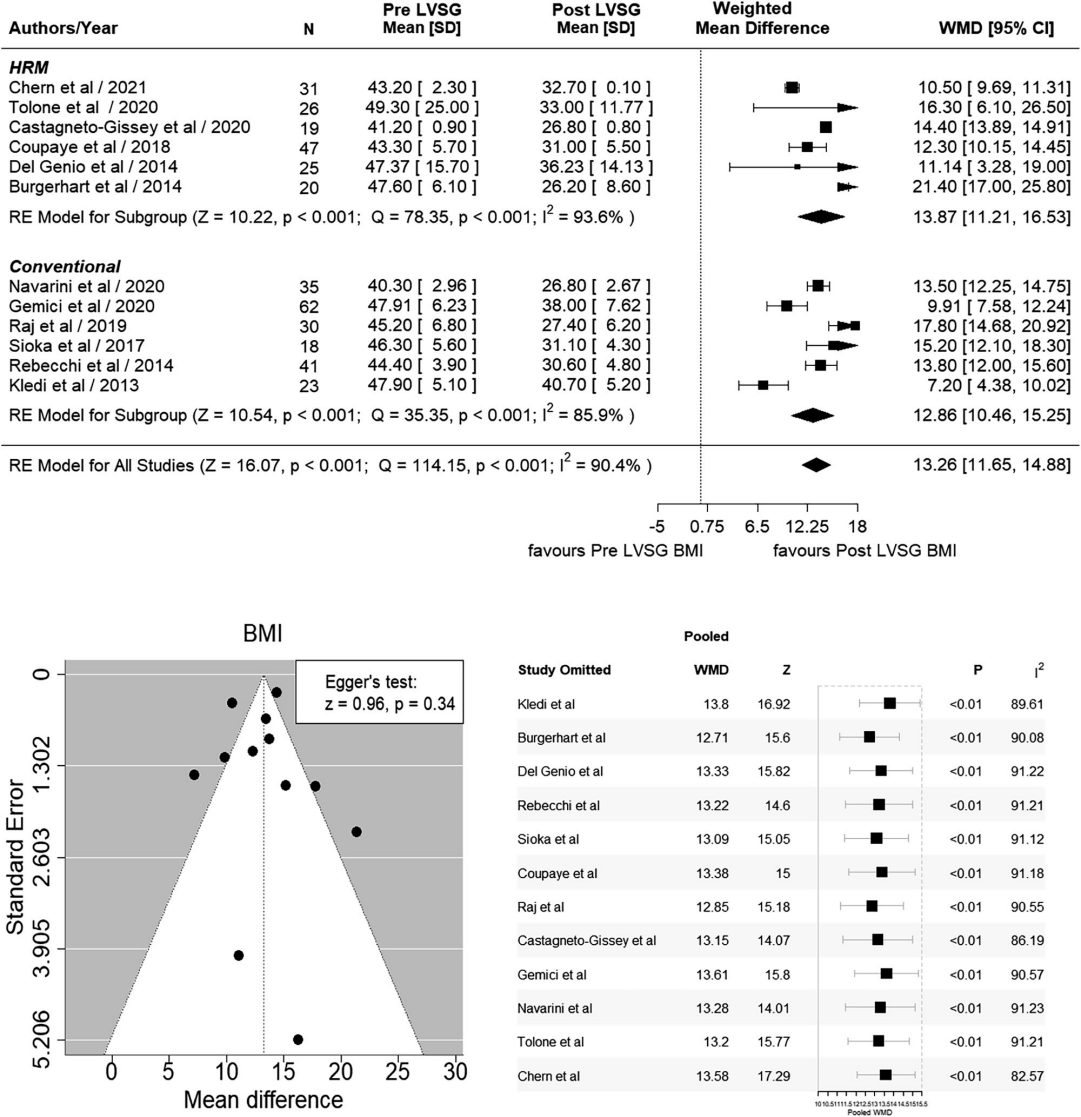
Forrest and funnel plots and sensitivity analysis of BMI (*r* = 0.1). **Forest plot (Top):** Significant reduction of 13.26 kg/m^2^ was observed in BMI for post LVSG compared to pre LVSG (WMD 13.26, 95% CI 11.65 to 14.88, *Z* = 16.07, *p* < 0.001). Significant difference between all studies (*Q* = 114.15, *p* < 0.001, ${I}^{2}$ = 90.4%). **Funnel Plot (bottom left):** The funnel plot was asymmetrical, but there was no publication bias demonstrated by Egger’s test (*p* = 0.34). **Sensitivity Analysis (bottom right):** The largest decrease in the ${I}^{2}$ value was observed when Chern et al. was omitted from our analysis. However, our results across multiple iterations i.e., when we removed one data point/individual study, did not significantly change the outcome, and the pooled WMD and heterogeneity (>80%) remained relatively unchanged, indicating that the overall conclusions are robust.

Nineteen studies [16–34] (prospective = 16 [16–18, 20–28, 30–33] retrospective = 3 [19, 29, 34] analyzing LESP and LESL ± 24 h pH were included in our meta-analysis (Table 2). A total of 668 patients were analyzed (*F* = 445, *M* = 131). The largest study from Brazil [23] included 73 patients, whereas the smallest study was conducted in Argentina [21] with 14 patients. The countries that contributed to these studies included Italy four studies [19, 20, 27, 32], France three studies [24, 29, 31], Brazil two studies [23, 30] Greece two studies [17, 22], and one each by Argentina [21], Australia [33], Chile [16], India [26], the Netherlands [18], Spain [25], Türkiye [28] and Peru [34]. Nine studies used HRM [18, 19, 24, 27, 31–34] and 10 used CM [16, 17, 20–23, 25, 26, 28] to assess LES physiology. Twelve studies [18–21, 24–28, 30, 33, 34] undertook 24 h pH studies to assess DMS. The postoperative follow-up period ranged from 6 weeks to 24 months. A significant reduction of 3.82 mm Hg in LESP was observed after LVSG compared to pre-LVSG patients based on 16 studies [16, 18–21, 23–28, 30–34] (WMD 3.82, 95% CI 1.74, 5.90; *p* < 0.001, *I*^2^ = 88.6%). Egger’s test failed to reveal any publication bias (*p* = 0.49) (Fig. 2). LESL did not reveal any significant difference between pre- and post-LVSG in nine studies [17, 19–21, 25, 26, 28, 31, 33] (WMD 0.05, 95% CI –0.15, 0.26; *p* = 0.625, *I*^2^ = 83.1%). However, when we performed leave out sensitivity analysis with the removal of Keldi’s et al. study [17] it showed significant decrease of 0.16 cm in LESL (WMD 0.16, 95% CI 0.00 to 0.32, *Z* = 2.00, *p* = 0.045) suggesting disproportionate influence of this study on the overall analysis (Fig. 3). Egger’s test did confirm publication bias (*p* = 0.06) with the inclusion of Keldi’s study. However, after removing it, although the funnel plot was still asymmetrical, there was no publication bias as per Egger’s test (*p* = 0.46) (Fig. 3). The DeMeester Score (DMS) showed a significant increase in 11.72 post LVSG vs pre LVSG based on 12 studies [18–21, 24–28, 30, 33, 34] (WMD –11.72, 95% CI –17.15 to –6.30; *p* < 0.001, *I*^2^ = 91.5%). Egger’s test confirmed the publication bias (*p* = <0.01) (Fig. 4). The BMI analysis revealed a significant reduction of 13.26 kg/m^2^ post-LVSG compared to pre-LVSG based on 12 studies [17–20, 22, 24, 26–28, 30, 32, 33] (WMD 13.26, 95% CI 11.65 to 14.88, *Z* = 16.07, *p* < 0.001). The funnel plot was asymmetrical, but there was no publication bias demonstrated by Egger’s test (*p* = 0.34). (Fig. 5). As Egger’s test demonstrated no publication bias for LESP, LESL (when Keldi’s study was excluded) and BMI, this confirms the robustness of our analysis, even when heterogeneity was evident from *I*^*2*^ values and visual inspection of forrest and funnel plots (Figs. 2–5). Furthermore, the results of our meta-analysis remained stable and consistent across all leave-one-out iterations, suggesting that the overall effect estimate is robust and not heavily influenced by any single study (Figs. 2–5).

**Table 2:** Salient features of cohort studies.

Authors	Country	Year	Study Type	Pts	M	F	Age	Pre LVSG BMI	Post LVSG BMI	Manometry	Motility disorders	Pre LVSG GERD	Pre LVSG PPIs	Post LVSG PPIs	Hiatal Hernia	Follow-up
				***N***	***N***	***N***	**Mean** **±** **SD (Range)**	**kg/m**^**2**^	**kg/m**^**2**^	**CM or HRM**	**%**	**%**	**%**	**%**	**%**	
**Braghetto et al.** [16]	Chile	2010	Prospective	20	3	17	37.6 ± 12.6 (23–55)	38.3 ± 3.47 (34–44)	28.2	CM	NA	NA	NA	NA	NA	6 Months, Mean ± SD
**Kledi et al.** [17]	Greece	2013	Prospective	23	12	11	38.5 ± 10.9	47.9 ± 5.1	40.7 ± 5.2	CM	NA	NA	NA	NA	NA	6 weeks, Mean ± SD
**Burgerhart et al.** [18]	The Netherlands	2014	Prospective	20	4	16	43 ± 12	47.6 ± 6.1	26.2 ± 8.6	HRM	NA	70	45	NA	25	3 Months, Mean ± SD
**Del Genio et al.** [19]	Italy	2014	Retrospective	25	7	18	42 (22–62)	46.1 (38–58)	34.7 (28–46)	HRM	40%	NA	NA	NA	0	13 (11–17) Months, Median (IQR)
**Rebecchi et al.** [20]	Italy	2014	Prospective	41	3	38	43.1 ± 10.5	44.4 ± 3.9	30.6 ± 4.8	CM	NA	19.5	19.5	18.9	7.3	24 Months with normal acid exposure, Mean ± SD
**Gorodner et al.** [21]	Argentina	2015	Prospective	14	1	13	42 ± 12	40 ± 6	NA	CM	7	50	NA	NA	NA	12 Months, Mean ± SD
**Sioka et al.** [22]	Greece	2017	Prospective	18	8	10	40.7 ± 8.1 (30–56)	46.3 ± 5.6 (38-58)	31.1 ± 4.3 (24–40)	CM	Hypertensive contractions	NA	NA	NA	NA	7 Months (6–15), Mean ± SD
**Valezi et al.** [23]	Brazil	2017	Prospective	73	18	55	40.2 (19–61)	41.1 (35–46)	NA	CM	No motility disorders	0	NA	NA	NA	12 Months, Mean ± SD
**Coupaye et al.** [24]	France	2018	Prospective	47	1	46	41.1 ± 9.4	43.3 ± 5.7	31.0 ± 5.5	HRM	12.70	36	17	28	8.5	12 Months, Mean ± SD, 16 +ve GERD, 31 No GERD
**Ruiz de Angulo et al.** [25]	Spain	2019	Prospective	26	12	14	45.27 ± 10.14	46.62 ± 5.11	NA	CM	NA	46	23	NA	15.3	12 Months, Mean ± SD
**Raj et al.** [26]	India	2019	Prospective	30	8	22	37.8 ± 11.5	45.2 ± 6.8	27.4 ± 6.2	CM	NA	0	0	NA	NA	6 Months, Non GERD patients
**Castagneto-Gissey et al.** [27]	Italy	2020	Prospective	19	4	15	41.6 ± 2.8	41.2 ± 0.9	26.8 ± 0.8	HRM	NA	10.5	10.5%	42.1%	NA	12 Months, Mean ± SD
**Gemici et al.** [28]	Türkiye	2020	Prospective	62	12	50	40.3 ± 10.6 (20–58)	47.91 ± 6.23	38 ± 7.62	CM	NA	NA	NA	NA	NA	3 Months, Mean ± SD
**Greilsamer et al.** [29]	France	2020	Retrospective	69	15	54	45.8 ± 9.8	47.6 ± 7.1	NA	HRM	11.40	36.2	NA	NA	17.4	32.1 ± 24.1 Months
**Navarini et al.** [30]	Brazil	2020	Prospective	35	6	29	40 ± 13	40.3 ± 4	26.8 ± 3.6	CM	NA	37	NA	NA	31	12 Months, Median ± IQR
**Quero et al.** [31]	France	2020	Prospective	23	6	17	36 ± 10	42.4 ± 5.8	32.8 ± 4.4	HRM	Increase IGP	43.4	NA	NA	NA	7.5 ± 2.9 Months, Mean±IC
**Tolone et al.** [32]	Italy	2020	Prospective	26	NA	NA	39 ± 12	42 (37–69)	30 (27–42)	HRM	NA	NA	NA	NA	0%	12 Months, Median ± IQR
**Chern et al.** [33]	Australia	2021	Prospective	31	11	20	45.7 ± 4.9	43.2 ± 2.3	32.7 ± 0.1	HRM	24%	80	15.8	31.6	NA	6 Months, Mean ± SD
**Poggi et al.** [34]	Peru	2023	Retrospective	66	NA	NA	NA	NA	NA	HRM	18.20%	28.4	NA	NA	NA	12–24 Months, Mean ± SD
				668	131	445										

*BMI* body mass index, *CM* conventional manometry, *F* female, *HRM* high resolution manometry, *kg/m*^2^ kilogram per square meter, *M* male, *LVSG* laparoscopic vertical sleeve gastrectomy, *NA* not available, *Pts* patients, *%* percentage.

## Discussion

According to the World Obesity Atlas 2023 report [35], “*38% of the global population is currently either overweight or obese, based on the WHO criteria. By 2035, the global prevalence of overweight and obesity is projected to reach 51%*”. Furthermore, by 2030, 78% of US adults are projected to be overweight/obese [36]. The cost of this global obesity pandemic is estimated to be more than four trillion US dollars of potential income in 2035 [35]. With the rapidly increasing global prevalence of obesity, bariatric surgery is routinely implemented to prevent the development of several chronic conditions and their associated complications. According to the International Federation for Surgery of Obesity and Metabolic Disorders (IFSO) 8TH Global Registry Report published in 2023 [37], 480,970 bariatric procedures were performed in 2021 and 2022. Of these, 60.4% were LVSG, 29.5% were laparoscopic Roux-en-Y gastric bypass (LRYGB), 4.3% were laparoscopic one-anastomosis gastric bypass (LOAGB), and 5.8% were others [37]. The revision rate for LVSG was 23.8%, the reasons for which were not specified. However, one can speculate that weight regain and GERD are the two most common reasons for revision surgery. Two recent meta-analyses comparing 5-year GER outcomes following LVSG and LRYGB have shown significantly worsened GERD, including the development of de novo GERD at 5-year following LVSG vs LRYGB (19.1% vs 3.4%) requiring either pharmacological or surgical intervention [5, 6]. Other recent publications reporting 5- to 10-year outcomes for LVSG vs LRYGB demonstrated ongoing worsened GERD outcomes for patients in the former compared to the latter group [38–40]. A number of possible explanations have been put forward to explain this phenomenon, which include (a) the presence of hiatal hernia (HH), which reduces LESP compared to those without HH (13 vs 8 mm Hg) [41] (b) the impact of sleeve shape on the degree of intraluminal (or intragastric) pressure, which is inversely proportional to the diameter of the gastric lumen post LVSG [42]; (c) the effect of resection of the fundus, which leads to decreased vasovagal reflex and complete elimination of physiological postprandial gastric relaxation, further increasing the intragastric pressure (IIGP) [5]; (d) poor surgical techniques resulting in sleeve stenosis, kinking, angulation, and/or cicatrization of the sleeve leading to IIGP [5, 6]; and (e) a more obtuse esophagogastric angle in the majority (78%) of patients following LVSG, which is associated with decreased intra-abdominal length and resting pressure of the LES [31]. However, assessing the effect of GERD after LVSG is challenging due to the lack of standardized reporting, varying definitions, and often lack of objective assessment with the EFTs [5, 6].

Fyke et al. [43] were the first to describe an HPZ at the human EGJ in 1956. Two further studies [44, 45] provided undeniable evidence for the presence of LES and its importance in preventing GER. However, what constitutes LES remains a source of contention. Liebermann-Meffert et al. [46] described the sling muscle fibers on the greater curvature of the stomach and the clasp muscle fibers on the lesser curvature, both within the gastric cardia, and considered them to be the major anatomic component within the HPZ [47]. Stein et al. [47] compared three-dimensional manometric pressure images with muscular thickness and architecture in the human LES and concluded that the LES is not a muscular ring, and the arrangement of muscular structures at the EGJ indicates that the gastric sling fibers at the greater curvature and the semicircular clasps at the lesser curvature are the anatomic correlates of the manometric LES in human beings and are important in maintaining EGJ integrity. Zifran et al. [48] recently studied the myoarchitecture of the LES and esophageal hiatus using optical sectioning microscopy. According to them, the circular muscle fibers at the lower end of the esophagus cross its dorsal surface, close to the angle of His, and continue as oblique muscle fibers on the anterior and posterior surfaces of the stomach (from the greater curvature to the lesser curvature). According to the authors, the spiral muscle fibers of the distal esophagus, crossing at the angle of His, will provide a circumferential squeeze at the lower end of the esophagus and act like a “noose” around the esophagus, providing a barrier against GER.

As mentioned above, LES therefore plays an important role in the pathophysiology of GERD, both pre- and post-LVSG. Several studies have investigated the relevance of manometry and 24-h pH study to gauge the impact of LVSG on LESP and LESL in patients with morbid obesity, an area that seems to have been under-researched in the pathophysiology of GERD post-bariatric surgery. To date, all cohort studies comparing pre- and post-LVSG esophageal function data have been underpowered because of the small sample size, which may reduce the chance of detecting a true effect of surgery on LES, leading to unreliable conclusions. Therefore, our aim was to pool data from several independent studies to provide a more precise estimate of the outcome of LVSG for LESP, LESL, and GERD. Thus, we analyzed the objective data on the anatomical changes at the EGJ/LES, which may result in a reduction in the total and/or abdominal LESL and lead to dynamic failure by affecting the LESP profile [49]. These changes can be attributed to iatrogenic injury of the sling fibers at the cardia while dissecting around the angle of His during the LVSG procedure [50, 51].

Zaninotto et al. [2] were the first to illustrate the importance of not only TL and intraluminal LESP but also of AL in maintaining the competence of the LES. The overall LESL in healthy individuals varies between 2.5 and 5.5 cm. Several studies have analyzed LESL and its association with GER [4, 52] and have shown that LESL is reduced in GERD. Therefore, we evaluated the impact of LVSG on LESP, LESL (TL), LESAL, and DMS.

### Lower esophageal sphincter abdominal length (LESAL)

Except for one study [31], none of the other 19 studies in our meta-analysis provided manometry data on LESAL. Our efforts to contact corresponding authors of several of these studies repeatedly via email were unsuccessful and therefore, we excluded this variable from our analysis.

### Lower esophageal sphincter total length (LESL)

Although 12 studies provided data on LESL both pre- and post-LVSG, only nine studies [17, 19–21, 25, 26, 28, 31, 33], three performed HRM [19, 31, 33], and six CM [17, 20, 21, 25, 26, 28] were analyzable. All HRM studies have consistently shown a significant decrease in LESL, and in one case, significantly [31]. In contrast, all CM studies have shown no profound changes in the LESL after LVSG. Meta-analysis of pooled data from these studies failed to show any significant anatomical changes in the LESL pre- and post-LVSG (Fig. 3). However, when we performed leave out sensitivity analysis with the removal of Keldi’s et al. study [17] it showed significant decrease of 0.16 cm in LESL (WMD 0.16, 95% CI 0.00 to 0.32, *Z* = 2.00, *p* = 0.045) suggesting disproportionate influence of this study on the overall analysis (Fig. 3). Moreover, we feel that the analytical disparity between HRM and CM is due to the fact that the former is far more accurate than the latter in recognizing LESL changes pre- and post-LVSG due to the distribution of closely placed pressure sensors (every 1 cm), which prevents loss of relevant information, leading to a more accurate measurement of LESL along with high resolution, which leads to greater reproducibility. The present analysis, based on a combination of CM and HRM data, failed to show any substantial anatomical reduction in LESL post-LVSG (if one does not exclude any studies) and therefore cannot be considered a contributing factor to GERD. We hope that future large-scale multicenter trials will provide more accurate information on the impact of LVSG on LESL using HRM data alone.

### Lower Esophageal Sphincter Pressure (LESP)

Next, we analyzed pre- and post-LVSG LESP manometry data from 16 studies [16, 18–21, 23–28, 30–34] (HRM = 8 [18, 19, 24, 27, 31–34] CM = 8 [16, 20, 21, 23, 25, 26, 28, 30]), as the data from the remaining three studies [17, 22, 29] were not analyzable. It was evident that post-LVSG, there was a dynamic failure of LES due to a decrease in LESP in all studies except for two [19, 32]. The pooled data from these studies showed a significant reduction in post-LVSG LESP of 3.82 mm Hg (Fig. 2). Subgroup analysis for both HRM and CM showed a similar significant reduction in LESP in post-LVSG patients (Fig. 2). Furthermore, when we applied sensitivity analysis, our results across multiple iterations did not significantly change the outcome and the pooled WMD and heterogeneity (>80%) remained relatively unchanged, indicating that the overall conclusions are robust.

LESP at rest is maintained between 10 and 30 mmHg in healthy individuals. Even if the LES is relaxed, the pressure remains slightly higher than that of the IGP to prevent reflux. Following LVSG, reduced LESP and increase in intragastric pressure (IIGP) due to removal of 80–90% of the stomach augments the condition for GERD, leading to worsening of GER symptoms or even producing de novo GERD. Yehoshua et al. [53] undertook volume and pressure assessments pre- and post-LVSG using an electronic barostat. According to these authors, the primary motor function of the stomach is to receive, store, and prepare food for digestion. This task is made possible by the accommodation reflex, which, through active relaxation of the gastric fundus, allows for a volume increase without an IIGP, thus enabling the stomach to accommodate large volumes during food intake. According to them, the distensibility of the total stomach and excised fundus was 10-fold higher than that of the gastric sleeve, providing for the first time conclusive evidence that the distensible region of the stomach is removed during LVSG, leading to IIGP. Mion et al. [42] undertook high-resolution impedance manometry postoperatively to evaluate the impact of LVSG on esophagogastric motility, particularly in patients with upper gastrointestinal symptoms. They concluded that IIGP occurred in 77% of patients after water swallow, and impedance reflux episodes were observed in 53% of patients with LVSG, especially those with GER symptoms and ineffective esophageal motility, and were more pronounced in sleeves with smaller volumes and diameters. In another prospective study, IIGP after water swallows was observed in 50% of LVSG patients with de novo GERD (49 mmHg) than in those without this complication (25 mmHg). Five studies [18, 24, 28, 31, 33] in our analysis provided some information on IGP; one study [18] showed a decrease in IGP at 3 months following LVSG, one showed no change in IGP post-sleeve [28], whereas three studies [24, 31, 33] showed significant IIGP. Therefore, it can be theorized that increased GERD in LVSG patients occurs because IIGP frequently exceeds LES resting pressure, which is already low owing to the impact of surgery, a perfect condition for GER.

### DeMeester Score (DMS)

We next analyzed DMS based on 24-h pH data on pre- and post-LVSG cohorts in each study to determine whether the combination of low LESP and IIGP would increase the risk of GERD. Twelve studies [18–21, 24–28, 33, 34] provided pre- and post-LVSG DMS. Almost every study showed an increased DMS after LVS. Meta-analysis showed a significantly increased DMS of 11.72 post-LVSG (Fig. 4). Once again, by applying sensitivity analysis, our results across multiple iterations did not significantly change the outcome, and the pooled WMD and heterogeneity (>80%) remained relatively unchanged, indicating that the overall conclusions are valid.

The increase in DMS is most likely due to low LESP and IIGP levels, which also contribute to an increase in the number of TLESRs, leading to either worsening of GERD or initiation of de novo GERD. Moreover, dynamic LES failure may further amplify GERD symptoms, particularly in cases of coexisting primary esophageal motility disorder (PEMD) and HH. Six [18, 21, 24, 29, 33, 34] of the included studies provided additional manometric data on PEMD, and five [18, 20, 24, 25, 29] on HH. The prevalence of PEMD varied from 11% to 40% in these studies, and that of HH from 8.5% to 31%. Jaffin et al. [54] were the first to bring to our attention the existence of PEMD in patients with morbid obesity, the prevalence of which was 61% in their cohort. Similarly, a few subsequent studies in patients with morbid obesity have shown a high incidence of PEMD based on manometry data [23, 55–57]. Several studies have also undertaken preoperative gastroscopy in patients with morbid obesity to assess the issue of HH and reflux esophagitis, the incidence of which varies from 5.4% to 52.6% [58, 59] and from 4.4% to 42% [60, 61], respectively. Mion et al. [42] have shown that patients with impedance reflux episodes had more frequent manometric abnormalities observed with proven GER symptoms such as low baseline EJG pressure and ineffective esophageal motility [62, 63]. Furthermore, manometric HH was also more frequently observed in patients with GERD. Our analysis has shown that a considerable number of patients have HH and PEMD [18, 24], which most likely exacerbates the risk of postoperative GERD and de novo GERD in LVSG patients.

Lastly, we analyzed the pre- and post-LVSG BMI losses across all the studies. The BMI analysis revealed a significant reduction of 13.26 kg/m^2^ post LVSG compared to pre-LVSG based on 12 studies [17–20, 22, 24, 26–28, 30, 32, 33] (WMD 13.26, 95% CI 11.65 to 14.88, *Z* = 16.07, *p* < 0.001) (Fig. 5). The asymmetric distribution of studies around the overall effect indicates the presence of publication bias (*Q* = 114.15, *p* < 0.001, ${I}^{2}$ = 90.4%). The largest decrease in the ${I}^{2}$ value was observed when Chern et al. study [33] was omitted from our analysis. However, our results across multiple iterations did not significantly change the outcome, and the pooled WMD and heterogeneity (>80%) remained relatively unchanged, indicating that the overall conclusions are reliable. Therefore, one can conclude that there is not necessarily a parallel reduction of GERD and weight loss post-LVSG. In fact, based on our meta-analysis, it is apparent that a substantial number of patients post LVSG seem to be experiencing either worsening or new-onset (de novo) GERD symptoms even in a short period of time.

Dupree et al. [64] analyzed 4832 LVSG patients with pre-existing GERD in 44.5% of the cohort. According to this study, 84.1% of patients continued to have GERD symptoms postoperatively, with only 15.9% demonstrating GERD resolution. Among the LVSG patients who did not demonstrate preoperative GERD, 8.6% developed de novo GERD postoperatively. The authors concluded that there is a large population at risk of potential adverse outcomes after LVSG, as this procedure is associated with LES anatomical and physiological changes that increase the risk of postoperative GERD. However, these conclusions were based on: (a) the subjective analysis of inpatient and outpatient follow-up data with no objective assessment, such as a 24-h pH study, and (b) the analysis involved only three years of data. There was certainly no mention of further longitudinal follow-up for these patients. Contrary to Dupree’s article, a recently published meta-analysis [6] scrutinizing 5 years of longitudinal data has shown a much higher improvement in GER symptoms between 40 and 60% in LVSG patients, which is most likely due to weight reduction. Even then, a significantly higher number of LVSG patients i.e., 19.1% compared to 3.4% LRYGB patients, reported worsened or de novo GERD at 5 years, requiring either pharmacological or surgical intervention [6]. Additionally, 6.25% of LVSG patients required surgical revision to manage their severe GERD symptoms. None of the patients who underwent LRYGB required any surgical intervention for GERD symptoms ^(6)^. A recent IFSO report [37] has similarly shown that the revision rate for sleeve gastrectomy was 23.8%, although the reasons for this were not provided. We feel that post-bariatric surgery, weight regain, and GERD would most likely be the two important reasons for revisional surgery.

## Limitations

There are a number of limitations to the present meta-analysis that need to be acknowledged. First, all the included studies to date were underpowered due to the small sample size, which may reduce the chance of detecting a true effect. Second, heterogeneity of data due to the variation in study outcomes between studies was observed, which may impact the certainty of evidence presented. However, variability among research findings is an inevitable reality and is due to a number of reasons which includes methodological discrepancies, patient characteristics and surgical experience to name but a view. We feel heterogeneity in our systematic review produces robust, generalizable evidence that can be effectively applied in real-world context to a much broader groups of patients. However, the results of our meta-analysis remained stable and consistent across all leave-one-out iterations, suggesting that the overall effect estimate is robust and not heavily influenced by any single study (Figs. 2–5). Additionally, Egger’s test demonstrated no publication bias for LESP, LESL (when Keldi’s study was excluded) and BMI, further confirming the robustness of our analysis. We therefore feel our results can be considered generalizable and our findings can be applied in a broader context. Third, the lack of data on some confounders, such as hiatus hernia and surgical technique, may impact the true incidence of GERD. Fourth, the inadequate quality of these studies was because they were either retrospective or prospective in nature with some missing data. Fifth, except for a few studies [20, 29, 34], the follow-up period was short, between 3 and 24 months, which is insufficient to accurately estimate the long-term incidence of GERD post-bariatric surgery. Lastly, there could be other unexplained factors that may have contributed to GERD in LVSG, such as an early surgical learning curve, different sizes of bougies, shape of the sleeve, and the proximity of the resection performed from the antrum, to name but a few.

## Conclusions

This is the first meta-analysis to analyze objective esophageal function data comparing pre- and post-LVSG, and it has conclusively demonstrated the detrimental effect of LVSG on LESP and DMS post-surgery despite significant loss of BMI. The consequences of this dynamic failure of LES are the development and/or worsening of GERD symptoms and/or development of de novo GERD in the short term [5], and erosive esophagitis [65], Barrett’s esophagitis [66], and esophageal adenocarcinoma [67] in the long term, the risk of which will continue to increase over the years, requiring close surveillance of these patients. A recent systematic review and meta-analysis [68] of more than 2000 patients who underwent LVSG has shown this to be the case. There was (a) a statistically significant increase in the use of PPIs and (b) there is 5.6% increase in the incidence of de novo BE which increased significantly in patients ≥10 years follow-up. It is therefore imperative that this subset of patients should be routinely screened for BE post-LVSG. Additionally, the authors also found a statistically significant increase in the incidence of GERD symptoms, EE, and HH post-LVSG, which may be contributing to the postoperative incidence of BE. It is therefore apparent that the cost of regular pharmacological intervention or requirement for revisional bariatric surgery will continue to climb and will have a negative financial impact on both patients and the healthcare system in the coming years. This has already been confirmed by the IFSO 8^th^ Global Registry Report [37]. All these findings should raise alarm among surgical fraternities and clinical decision makers. It is hoped that appropriate patient selection prior to bariatric surgery using EFTs will advance our understanding of LES physiology and any associated PEMD in patients with morbid obesity, thereby preventing inappropriate bariatric procedures in the first place and optimizing the choice of revisional bariatric procedures if necessary.

## Data Availability

Research data involving various publications is available from numerous electronic databases (Medline, PubMed, EMBASE, Cochrane Register of Systematic Reviews, Science Citation Index, and journal websites).
